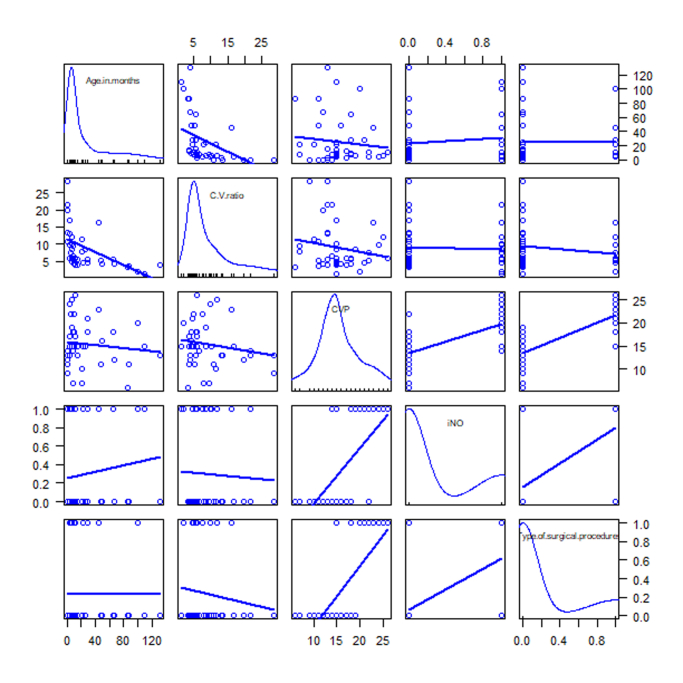# Morphology-Based Risk Analysis of Catheter-related Thrombus After Pediatric Cardiac Surgery

**DOI:** 10.1016/j.atssr.2024.01.013

**Published:** 2024-02-16

**Authors:** Hisataka Nozawa, Tomomi Fujimura, Tomosato Yamagata, Ayumi Kunikata, Kaname Uchida, Hidehito Ota, Hironori Ebishima, Kenichiro Hayashi, Hikoro Matsui

**Affiliations:** 1 Department of Pediatrics, The University of Tokyo Hospital, Tokyo, Japan

## Abstract

**Background:**

Understanding the development of central venous catheter-related thrombus (CVCRT) is vital for the prevention of adverse events caused by thrombi after cardiac surgery in children. However, the risks associated with CVCRT remain controversial. This study analyzed the risk factors of CVCRT based on a detailed evaluation of its morphometric features and severity.

**Methods:**

Patients aged <15 years who underwent catheter insertion into the internal jugular vein for cardiac surgery were included, and those receiving extracorporeal membrane oxygenation were excluded. The clinical data of the eligible patients, including the ultrasound CVCRT images and the ratio of the catheter occupying the internal jugular vein area (C/V ratio) by reassuming the images, were consistently collected. Logistic regression analysis using clinical factors was performed for the 2 groups divided according to morphologic severity.

**Results:**

Forty-seven patients were included in the study. CVCRT developed in 38 patients. Five graded types, ranging from wall-localized small thrombi to complete occlusion of the vein, were detected, and those who developed sheath-like thrombus were classified in to the severe group. Patients in the severe group were significantly younger and had higher C/V ratios. There were no significant differences in the surgical procedure, its difficulty, or postoperative severity score. Logistic regression analysis revealed the C/V ratio as the sole significant risk factor (odds ratio, 1.120; 95% CI, 1.01-1.24; *P* = .036).

**Conclusions:**

Our findings show the clinical implications of thrombus evaluation and morphologic classification to properly assess the risk factors of CVCRT in children with heart disease.

## Graphical abstract

**Figure:**
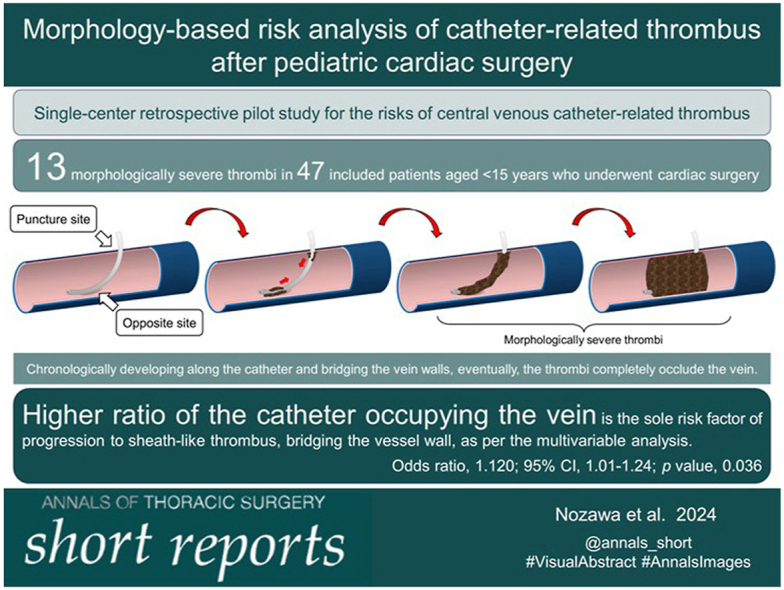

**In Short**

▪ It is important to consider the severity of the thrombus according to morphologic classification to properly assess the risk factors for central venous catheter-related thrombus after cardiac surgery in children.
▪ Using tomographic images obtained in real-time by ultrasonography may be effective in the evaluation of thrombus severity, in addition to the planar findings of stenosis rate on angiographic long-axis images.

Central venous catheter-related thrombus (CVCRT) develops in the perioperative period and causes a variety of critical adverse events in children with congenital heart disease (CHD), including severe organ embolisms, reduced central venous routes for catheterization or further cardiac surgeries, and unstable drug administration by catheter obstruction in critical patients after surgery.^1^ The identification of risk factors for the CVCRT is key to its prevention and treatment while exact clinical risk factors in children with CHD are still under discussion.

Understanding the morphologic severity of CVCRT or the physical relationship between the vessels and catheters is important for investigating the developmental process of CVCRT because these factors can directly cause stasis and turbulence around the central venous catheter (CVC). Thrombus formation is accelerated by congestion and turbulence of blood flow caused by inadequate clearance between the catheter and vessel due to high CVC occupation of the vein, especially in children with a small vascular diameter.^2^ However, few studies have focused on the morphologic aspects for the assessment of risk factors of CVCRT in children with CHD, and studies regarding the structural relationship between the vessel and catheter are still lacking. This study aimed to extract accurate risk factors for the development of CVCRT in children with CHD by including detailed morphologic characteristics of CVCRT.

## Patients and Methods

We investigated consecutive patients aged <15 years who underwent CVC insertion into the internal jugular vein (IJV) and were admitted to the pediatric intensive care unit of the University of Tokyo Hospital for postoperative care after cardiac surgery between January and June 2022. We excluded patients who received extracorporeal membrane oxygenation immediately after surgery because of their need for large amounts of anticoagulation therapy (ACT).

We further collected the clinical data of each patient, including background characteristics, perioperative data, and the size of the CVC, from electronic medical records. The endpoint was defined as the collection of perioperative data at the time of CVC removal. We also collected follow-up data of patients with CVCRT, including ACT for CVCRT and presence of residual thrombus. The CVC was preoperatively placed by a pediatric intensivist or anesthesiologist. One of 2 types of catheters (Argyle Fukuroi SMAC plus, 15G, Cardinal Health; ARROW Pediatric Three-Lumen CVC, 4Fr, Teleflex Incorporated) was chosen by the discretion of the operating physician.

**Supplemental Figure 1:**
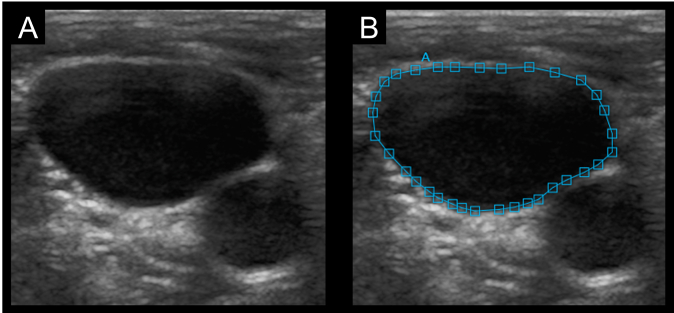


To investigate the relationship between catheter size and CVCRT, we analyzed the area of the IJV using ultrasound images obtained immediately after CVC removal and calculated the ratio of the cross-sectional area of the catheter to the IJV (C/V ratio). Supplemental Figure 1 shows an example of an actual measurement. If the IJV was completely occluded by a thrombus, it was judged as unmeasurable. We collected ultrasonographic CVCRT images of eligible patients from the electronic medical records, and classified the obtained images into 5 categories based on the classical classification of thrombi along with CVC by contrast radiography, as described by Bennegård and associates.^3^ We then categorized the patients to mild and severe groups according to the morphologic features of the thrombus. All images were captured using a high-resolution linear probe with an ultrasound machine to obtain detailed images of the thrombus and IJV (L15-7io linear probe, CX50, or EPIC elite ultrasound machine, Koninklijke Philips N.V.).

Descriptive statistics are expressed as median (interquartile range) for continuous data or as absolute frequencies and percentages for categorical data. The Mann-Whitney U and Fisher’s Exact tests were used for bivariate comparisons between the 2 groups. To examine the association between the C/V ratio and thrombus formation, we performed a binary logistic regression analysis (statistical significance was set at *P* < .05; see Supplemental Material for details). Patients with complete occlusion of the IJV were excluded from the analysis because the IJV area could not be measured. All analyses were performed using R version 4.0.3 (The R Foundation for Statistical Computing).

## Results

**Figure 1:**
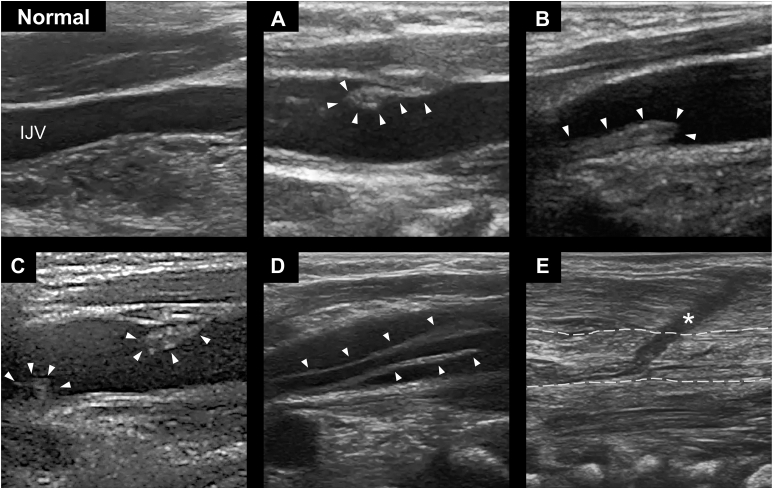
Morphologic classification of thrombus in the internal jugular vein (IJV). All images are captured with the patient's heart on the left side and head on the right side. (Normal) No thrombi. Scanning within the vessel, which has a low or no echogenic zone bounded by the vessel wall, shows complete absence of a structure equivalent to the echogenicity of the soft tissue. (A) Type A thrombus localized at the puncture site (white arrowhead). (B) Type B mural thrombus confined to the contralateral side of the puncture site (white arrowhead). (C) Type C wall thrombus on both sides of the puncture site and contralateral side (white arrowhead). (D) Type D thrombus bridging the vessel wall from the puncture site to the contralateral side, forming a Katana sheath-like structure (white arrowheads). (E) Complete occlusion of the vessel by thrombus (type E). The white dotted line indicates the vessel lumen. Asterisk (∗) indicates catheter passage through the subcutaneous tissue.

**Figure 2:**
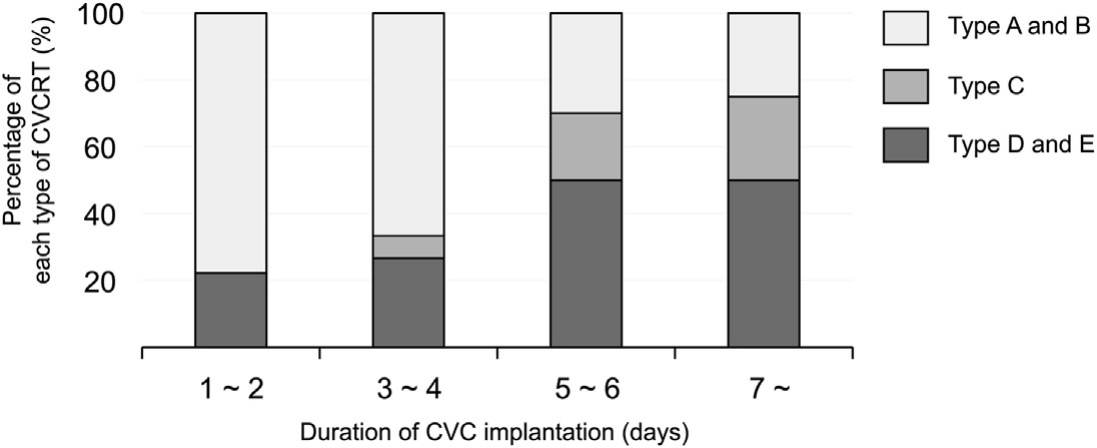
Shifting percentage of each type of central venous catheter-related thrombus (CVCRT) according to the duration of central venous catheter (CVC) implantation. The frequencies of types A and B are concentrated in the catheter implantation period of 2 days or less. As the duration increases, the frequencies of types C, D, and E increases.

During the study period, 48 patients were included in the study. One patient who underwent extracorporeal membrane oxygenation immediately after surgery was excluded; thus, 47 patients were eligible. Table 1 shows the demographic data, and Figure 1 demonstrates the morphologic classification of the thrombus into 5 categories. Of the 47 patients, CVCRT was identified in 38, which could be assigned to one of the following classifications: thrombus confined to the vessel wall (types A, B, or C), sheath-like thrombus bridging the vessel wall (type D), or complete occlusion of the vessel lumen (type E). Figure 2 shows the incidence of each type of CVCRT according to the number of days since CVC placement. Type E, the severest category of CVCRT, was identified in 2 patients. Eleven patients required ACT, 10 of whom were followed for residual thrombus; types D and E tended to be treatment-resistant with residual thrombus. No patients developed organ embolization due to CVCRT, although 2 patients who received ACT had bleeding complications.

**Table 1:** Descriptive Demographic Data of the Included Patients

Baseline Characteristics (N = 47)	Mild (n = 34)	Severe (n = 11)	CO (n =2)
Male	21 (61.8)	4 (36.4)	0 (0)
Age, mo	13.0 (8.0-40.8)	5.0 (0.0-6.5)	0 (0-0)
Type of surgical procedure			
Glenn or Fontan	8 (23.5)	3 (27.3)	0 (0)
STAT category 4 to 5	5 (16.1)	5 (50.0)	1 (50)
C/V ratio, %	5.8 (4.3-9.6)	12.0 (6.2-18.3)	NA
Duration of CVC implantation, d	3 (2-4)	5 (4-6)	3 (2-4)
VVR score	13.9 (4.5-19.2)	17.3 (10.1-24.8)	20.3 (20.1- 20.5)
Nonprophylactic ACT	6 (17.6)	3 (27.3)	0
Platelet count, × 10^4^ /μL	21.5 (16.4-26.4)	33.4 (24.0-46.5)	26.4 (36.4-16.4)
APTT, s	33.4 (30.2-42.2)	46.4 (34.7-56.2)	43.8 (43-44.5)
AT on postoperative day 1, %	88.5 (82.0-92.0)	76.5 (70.3-83.5)	66.5 (50-83)

Anticoagulation strategy for CVCRT (N = 11)	Value
Drugs of ACT, n	
Heparin	10
Warfarin	1
Rivaroxaban	7
ACT duration, d	65.5 (37-102.5)

Follow-up Data in Patients on ACT (10 of 11 cases)	Types A, B, and C	Type D	Type E
Follow-up rate, n/N (%)	5/5 (100)	3/4 (75)	2/2 (100)
Residual clot in follow-up cases, n/N (%)	1/5 (20)	1/3 (33.3)	2/2 (100)
Organ embolisms associated with CVCRT, events		0	
Adverse events of ACT, events			
Bleeding		2	

Values are presented as n (%) or median (interquartile range), unless otherwise noted. Mild group includes the cases without central venous catheter-related thrombus (CVCRT) or with types A, B, and C. Severe group falls into type D and complete occlusion (CO) group into type E.

ACT, anticoagulation therapy; APTT, activated partial thromboplastin time; AT, antithrombin; CVC, central venous catheter; C/V ratio, ratio of the catheter occupying the internal jugular vein area; STAT, The Society of Thoracic Surgeons-European Association for Cardiothoracic Surgery; VVR, vasoactive ventilation-renal score.

Based on morphologic classification, we categorized type D as severe and cases with no thrombus and types A, B, and C as mild. Table 2 and Supplemental Table 1 show the descriptive characteristics of the eligible patients and the results of the bivariate analysis between the 2 groups. The severe group demonstrated considerably fewer size-related factors, including age (in months) and greater C/V ratio than the mild group. There were no significant differences between the 2 groups regarding the surgical procedure, difficulty, or postoperative severity score. According to the results of the univariate analysis, multivariable analysis was performed with the C/V ratio, age, and surgical procedure as independent factors, and the occurrence of severe CVCRT as a dependent factor. The C/V ratio was the only significant risk factor for the development of severe CVCRT (Supplemental Figure 2, Table 2, Supplemental Table 2).

**Table 2:** Results of the Single Regression and Binary Logistic Regression Analyses

Single Regression Analysis	Mild (n = 34)	Severe (n = 11)	*P* Value
Male	21 (61.8)	4 (36.4)	.18
Age, mo	13.0 (8.0-40.8)	5.0 (0.0-6.5)	.011
Glenn or Fontan	8 (23.5)	3 (27.3)	>.99
STAT category 4 to 5	5 (16.1)	5 (50.0)	.17
C/V ratio, %	5.8 (4.3-9.6)	12.0 (6.2-18.3)	.03
Duration of CVC implantation, d	3 (2-4)	5 (4-6)	.01
VVR score	13.9 (4.5-19.2)	17.3 (10.1-24.8)	.22
Nonprophylactic ACT	6 (17.6)	3 (27.3)	.67
Platelet count, × 10^4^ /μL	21.5 (16.4-26.4)	33.4 (24.0-46.5)	.003
APTT, s	33.4 (30.2-42.2)	46.4 (34.7-56.2)	.05
AT on postoperative day 1, %	88.5 (82.0-92.0)	76.5 (70.3-83.5)	.04

Binary Logistic Regression Analysis	Odds Ratio	95% CI	*P* Value
Initial value of each statistic			
(Intercept)	0.19	0.03-1.25	.09
Glenn or Fontan	2.04	0.36-11.5	.42
C/V ratio	1.09	0.97-1.23	.16
Age, mo	0.97	0.93-1.02	.28
Statistics after variable selection process by stepwise method			
(Intercept)	0.11	0.03-0.42	.001
C/V ratio	1.12	1.01-1.24	.04

Values are presented as n (%) or median (interquartile range), unless otherwise noted.

ACT, anticoagulation therapy; APTT, activated partial thromboplastin time; AT, antithrombin; CI, confidence interval; CVC, central venous catheter; C/V ratio, ratio of the catheter occupying the internal jugular vein area; STAT, The Society of Thoracic Surgeons-European Association for Cardiothoracic Surgery; VVR, vasoactive ventilation-renal score.

## Comment

To begin with, we found the spatial extent of thrombus according to morphologic classification by high-resolution echography, visualizing the development of CVCRT in detail over time. When the catheter is placed for a longer duration, smooth muscle and endothelial cells gradually proliferate and coat the surface around the catheter after thrombus formation, followed by the formation of sheath-like fibrosis around the catheter, eventually occluding the vein with further thrombus formation.^4^ Our findings also demonstrated the chronological progression of thrombus growth from a wall thrombus to a shape along the catheter, eventually progressing to complete occlusion that is the most severe form of CVCRT. Furthermore, according to the follow-up data, morphologically severe CVCRT may be resistant to anticoagulants, suggesting a certain relationship between the clinical severity and response to ACT.

Categorizing the morphologic severity of the thrombus is key to the correct selection and analysis of risk factors for CVCRT. Although many reports have described risk factors for CVCRT in CHD, no definitive risk factor has been identified until now.^5^^,^^6^ This is because previous studies uniformly included and analyzed CVCRT that would have very different clinical contributions in the same group. Our study demonstrated that the C/V ratio is only a surrogate marker for CVCRT by classifying CVCRT according to morphologic severity and analyzing the geometric representation of the effect of CVC on the vessel. This C/V ratio is directly related to Virchow's triad, which consists of anatomical or physical factors that generate abnormal blood flow.^7^ In contrast, our study also revealed that there are still limitations in using age to analyze multiple intermediary factors. Although some reports have shown that age could be a possible significant factor for CVCRT formation in children,^8^ many pediatric patients with cardiac disease, especially severe cases, have generally small body sizes compared with their age.

This study has some limitations. First, this was a small single-center retrospective study. Second, because few imaging follow-ups and evaluations were performed in most cases, it was not possible to determine whether residual CVCRT was clinically problematic or relevant. Third, the CVCRT evaluation was performed after CVC extraction, which was not simultaneous with clot formation. Finally, type E cases, which are predicted to have the greatest influence on CVCRT development, were excluded from the 2-group analysis because the vein diameter could not be measured due to complete occlusion; in addition, their incidence was very low.

In conclusion, it is important to consider the severity of the thrombus according to the morphologic classification to properly assess the risk factors for CVCRT in children with CHD.

## Figures and Tables

**Supplemental Figure 2:**