# Supplementary material for: Morphology-Based Risk Analysis of Catheter-related Thrombus After Pediatric Cardiac Surgery
Source: Ann Thorac Surg Short Rep. 2024 Feb 16;2(3):380–4. doi: 10.1016/j.atssr.2024.01.013 (PMC11708739; doi:10.1016/j.atssr.2024.01.013)
Supplement: Appendix [file mmc1.docx]

**Appendix**

Methods

Statistical analysis

On CVCRT formation, to examine the causal relationship between the C/V ratio and clinical risk factors which may influence the diameter and morphology of veins, we performed a binary logistic regression analysis.

First, based on a comprehensive review of the prior literature and clinical perspectives, we extracted age in months, type of surgical procedures (Glenn and Fontan procedures or the others), inhalation nitric oxide therapy, and maximum value of CVP as confounders that affect both the morphological variation of IJV and thrombus formation.

Second, among these, inhalation nitric oxide therapy and maximum value of CVP were excluded because they were highly dependent on type of surgical procedures, and were judged to be highly collinear with each other (Supplemental Figure 2).

Finally, we performed a binary logistic regression analysis using a backward stepwise method with Akaike’s information criterion (p values less than 0.05 were considered significant), using the C/V ratio, age in months, and type of surgical procedures as explanatory variables, and set the aforesaid two groups as a response variable.

Results

Supplemental Table 1 Patient characteristics and the results of single regression analysis

| Variables | Control  (N = 34) | Case  (N = 11) | *p* value |
| --- | --- | --- | --- |
| **Patients’ background data** |  |  |  |
| Male, (%) | 21 (61.8) | 4 (36.4) | 0.176 |
| Age, months | 13.0 (8.0-40.8) | 5.0 (0.0-6.5) | 0.011 |
| Body weight, kg | 8.9 (6.8-12.2) | 5.6 (3.0-7.7) | 0.014 |
| Hight, cm | 72.8 (65.3-87.0) | 59.5 (48.0-67.1) | 0.012 |
| History of thrombosis, (%) | 2 (5.9) | 1 (9.1) | 1.000 |
|  |  |  |  |
| **Operation data** |  |  |  |
| The type of surgical procedure |  |  | 1.000 |
| Glenn or Fontan, (%) | 8 (23.5) | 3 (27.3) |  |
| Others | 26 (76.5) | 10 (72.7) |  |
| STAT category 4 to 5, (%) | 5 (16.1) | 5 (50.0) | 0.169 |
| Pump duration, minutes | 149 (106-209) | 147 (106-197) | 0.713 |
| Total amounts of blood products, ml/kg |  |  |  |
| RBC | 0.0 (0.0-2.4) | 0.0 (0.0-3.7) | 0.812 |
| FFP | 13.7 (10.5-18.2) | 9.0 (7.6-13.3) | 0.142 |
| PC | 3.7 (0.0-7.5) | 0.0 (0.0-6.7) | 0.597 |
|  |  |  |  |
| **CVC and vein data** |  |  |  |
| Mean vein area, mm^2^ | 38.5 (24.8-51.5) | 19.0 (12.5-38.0) | 0.039 |
| C/V ratio, % | 5.8 (4.3-9.6) | 12.0 (6.2-18.3) | 0.032 |
| Left side insertion, (%) | 2 (5.9) | 1 (9.1) | 1.000 |
| Duration of CVC implantation, days. | 3 (2-4) | 5 (4-6) | 0.013 |
|  |  |  |  |
| **Postoperative data** |  |  |  |
| Duration of intubation, days | 2 (1-3) | 4 (2-6) | 0.065 |
| VVR score | 13.9 (4.5-19.2) | 17.3 (10.1-24.8) | 0.217 |
| CVP, mmHg | 15 (13-18) | 15 (14-20) | 0.559 |
| Steroid, (%) | 17 (50.0) | 6 (54.5) | 1.000 |
| Neuromuscular blockade, (%) | 6 (17.6) | 4 (36.4) | 0.228 |
| Nitric oxide inhalation therapy, (%) | 10 (29.4) | 5 (45.5) | 0.464 |
| Fluid concentration of calcium, mEq/L | 23.4 (11.7-39.0) | 39.0 (28.7-46.8) | 0.192 |
| Nonprophylactic anticoagulation, (%) | 6 (17.6) | 3 (27.3) | 0.666 |
| Heparin, (%) | 6 (17.6) | 3 (27.3) | 0.666 |
| Warfarin, (%) | 2 (5.9) | 3 (27.3) | 0.085 |
| Total amounts of blood products, ml/kg |  |  |  |
| RBC | 0.0 (0.0-9.6) | 0.0 (0.0-9.0) | 1.000 |
| FFP | 22.7 (15.2-28.4) | 24.8 (13.0-31.5) | 0.905 |
| PC | 4.0 (0.0-9.4) | 0.0 (0.0-11.3) | 0.584 |
| Laboratory data |  |  |  |
| Hematocrit, % | 44.8 (41.5-49.2) | 48.2 (46.7-50.3) | 0.057 |
| Platelet count, × 10^4^ /µL | 21.5 (16.4-26.4) | 33.4 (24.0-46.5) | 0.003 |
| PT-INR | 1.24 (1.17-1.31) | 1.22 (1.17-1.35) | 0.884 |
| APTT, second | 33.4 (30.2-42.2) | 46.4 (34.7-56.2) | 0.054 |
| Antithrombin of postoperative day 1 | 88.5 (82.0-92.0) | 76.5 (70.3-83.5) | 0.039 |
| Blood glucose, mg/dL. | 169 (148-188) | 203 (168-243) | 0.091 |
| Complication |  |  |  |
| Infection, (%) | None | None | NA |

APTT, activated partial thromboplastin time; CVC, central venous catheter; CVP, central venous pressure; C/V ratio, ratio of the catheter occupying the IJV area; FFP, fresh frozen plasma; PC, platelet concentrate; RBC, red blood cell; STAT, The Society of Thoracic Surgeons-European Association for Cardio-Thoracic Surgery; VVR, vasoactive ventilation-renal score

Supplemental Table 2 Results of the binary logistic regression analysis

|  | VIF | Odds ratio | 95% CI | *p* value |
| --- | --- | --- | --- | --- |
| Initial value of each statistic | | | | |
| (Intercept) |  | 0.19 | 0.03-1.25 | 0.09 |
| Type of surgical procedures | 1.1 | 2.04 | 0.36-11.5 | 0.42 |
| C/V ratio | 1.2 | 1.09 | 0.97-1.23 | 0.16 |
| Age in months | 1.2 | 0.97 | 0.93-1.02 | 0.28 |
| AIC value | 49.74 | | | |
| Statistics after variable selection process by stepwise method | | | | |
| (Intercept) |  | 0.11 | 0.03-0.42 | 0.001 |
| C/V ratio |  | 1.12 | 1.01-1.24 | 0.04 |
| AIC value | 48.06 | | | |

AIC, Akaike information criterion; CI, confidence interval; C/V ratio, ratio of the catheter occupying the IJV area; VIF, variance inflation factor

Supplemental Figure 1. Method of measuring the cross-sectional area of the internal jugular vein

A: A short-axis image of the vein is continuously scanned from the catheter insertion site to as central as possible.

B: Average of the maximum and minimum vein areas of axial view obtained by tracking the limbus of the vein at 10 or more points is calculated.

Supplemental Figure 2. Scatterplot matrix of extracted factors

Inhalation nitric oxide therapy (iNO) and maximum value of CVP (CVP) were highly dependent on type of surgical procedures, and were judged to be highly collinear with each other.
